# Clinical potential and challenges of using genetically modified cells for articular cartilage repair

**DOI:** 10.3325/cmj.2011.52.245

**Published:** 2011-06

**Authors:** Henning Madry, Magali Cucchiarini

**Affiliations:** 1 Experimental Orthopaedics and Osteoarthritis Research, Saarland University Medical Center and Saarland University, Homburg/Saar, Germany; 2 Department of Orthopaedic Surgery, Saarland University Medical Center and Saarland University, Homburg/Saar, Germany

## Abstract

Articular cartilage defects do not regenerate. Transplantation of autologous articular chondrocytes, which is clinically being performed since several decades, laid the foundation for the transplantation of genetically modified cells, which may serve the dual role of providing a cell population capable of chondrogenesis and an additional stimulus for targeted articular cartilage repair. Experimental data generated so far have shown that genetically modified articular chondrocytes and mesenchymal stem cells (MSC) allow for sustained transgene expression when transplanted into articular cartilage defects in vivo. Overexpression of therapeutic factors enhances the structural features of the cartilaginous repair tissue. Combined overexpression of genes with complementary mechanisms of action is also feasible, holding promises for further enhancement of articular cartilage repair. Significant benefits have been also observed in preclinical animal models that are, in principle, more appropriate to the clinical situation. Finally, there is convincing proof of concept based on a phase I clinical gene therapy study in which transduced fibroblasts were injected into the metacarpophalangeal joints of patients without adverse events. To realize the full clinical potential of this approach, issues that need to be addressed include its safety, the choice of the ideal gene vector system allowing for a long-term transgene expression, the identification of the optimal therapeutic gene(s), the transplantation without or with supportive biomaterials, and the establishment of the optimal dose of modified cells. As safe techniques for generating genetically engineered articular chondrocytes and MSCs are available, they may eventually represent new avenues for improved cell-based therapies for articular cartilage repair. This, in turn, may provide an important step toward the unanswered question of articular cartilage regeneration.

In the early 1990s, Christopher H. Evans proposed and implemented the idea of using genetically modified cells for the treatment of arthritis (1). When synovial cells modified ex vivo via retroviral vector gene transfer were injected into the metacarpophalangeal joints of patients with rheumatoid arthritis, an interleukin-1 receptor antagonist (IL-1Ra), the active transgene product, was successfully expressed and biologically active (2). This study confirmed the feasibility and laid the basis for the clinical application of genetically modified cells to human joints (3). Since then, considerable progress has been made toward our understanding of the biological and technological requirements for a successful transplantation of genetically modified cells overexpressing secreted therapeutic proteins in vitro and in vivo.

An articular cartilage defect is an area of damaged or missing cartilage. Although often caused by acute trauma, cartilage defects may also occur as a result of osteoarthritis, osteonecrosis, osteochondritis dissecans, and other pathologies (4). While traumatic defects usually are well defined and surrounded by normal articular cartilage, defects caused by osteoarthritis are often ill-defined, large, and surrounded by osteoarthritic tissue of variable quality. When restricted to the articular cartilage, the defects are termed chondral and when they reach into the subchondral bone, they have an osteochondral nature. In contrast with the very limited repairability of chondral defects (that are only partly repopulated by cells from the synovial membrane), osteochondral defects are filled with a bone marrow clot that serves as a basis for the cartilaginous repair tissue that is formed by pluripotent undifferentiated mesenchymal stem cells (MSC) arising from the bone marrow. Such articular cartilage defects may lead to the development of osteoarthritis. Since degenerative joint diseases affect more than a third of the world population and disorders of the articulations, in general, account for more than half of all chronic conditions in persons aged 60 years and over, optimized treatment strategies for articular cartilage lesions are of a high socio-economic importance (5).

Symptomatic cartilage defects require surgical treatment. Treatment options include marrow-stimulating techniques such as microfracture, Pridie drilling and abrasion arthroplasty, autologous chondrocyte transplantation, and the transfer of both autologous or allogeneic osteochondral transplants (6). Autologous chondrocyte transplantation involves the isolation and culture of articular chondrocytes that are subsequently implanted in conjunction with a supportive biodegradable matrix into the articular cartilage defect. Several recent randomized controlled clinical studies confirmed the potential of autologous chondrocyte transplantation to repair large chondral defects (6). These promising data have laid a clinical basis for the experimental transplantation of genetically modified cells. In the past decades, considerable progress has been made to establish the scientific foundation for the use of such modified cells. In particular, articular chondrocytes and, more recently, MSCs have been genetically modified using nonviral or viral methods. When used in model systems of articular cartilage defects, these cells provide chondrogenic factors selectively stimulating chondrogenic processes, such as precursor cell and chondrocyte proliferation and/or the synthesis of extracellular matrix components like type-II collagen and proteoglycans. Moreover, in small and large animal models, transplantation of genetically modified cells has resulted in significant structural improvements in the repair tissue. Genetic modification of cells may therefore improve their therapeutic potential by selectively delivering biologically active compounds to a site of an articular cartilage defect. Moreover, genetically modified cells may serve the dual role of providing a repair stimulus to the host tissue and a target cell population that is capable of chondrogenesis (7).

The aim of this review is to outline options for genetic manipulation of target cells for articular cartilage repair, primarily articular chondrocytes and MSCs, and the current progress in adapting strategies based on chondrocyte transplantation for the delivery of genetically-modified cells for clinical use to treat focal articular cartilage defects and osteoarthritis.

## Gene transfer in precursor cells and articular chondrocytes

In the past two decades, articular chondrocytes and MSCs have been successfully transplanted into articular cartilage defects in patients, with the aim of improving articular cartilage repair. MSCs display some advantages compared with differentiated cells that make them attractive targets for manipulation in the goal of cartilage and bone regeneration. They can be easily isolated in a noninvasive and abundant manner from various tissues like the bone marrow, bone, adipose tissue, muscle, synovium, periosteum, and perichondrium (7)). They have a potential for self-renewal and to give rise to different tissues under adapted stimuli (cartilage, bone, adipose tissue). Also, their multilineage potential can be maintained in culture over time, in contrast to chondrocytes that lose their phenotype in such conditions. Remarkably, MSCs have immunosuppressive properties that might permit allo- and xenotransplantation. Preliminary clinical studies have shown the benefits of transplanting progenitor cells in human cartilage defects for instance, allowing for the formation of a stable repair tissue of fibrocartilaginous quality. As these cells, in particular articular chondrocytes, are relatively permissive to a variety of gene transfer systems, including nonviral and viral methods, researchers and clinicians have begun to evaluate the effect of gene transfer using a range of potentially therapeutic gene products. Other potential target cells for genetic modification and subsequent transplantation include cells from the tissues surrounding the joint such as cells of the synovial lining, bone, muscle, tendons, ligaments, and menisci.

Gene transfer using various reporter gene sequences has been successfully achieved in all these cell types by application of either nonviral compounds (8-20) or of viral vectors based on adenoviruses (8,11,21-38), retroviruses (8,11,21,25,39-44), and recombinant adeno-associated viruses (rAAV) (11,45-66). The latter vectors are currently the most potent gene delivery vehicles available, as they can efficiently and durably transduce articular chondrocytes (45,47,48,54-57), MSCs (45,46,58,59), synoviocytes (49-53), and other relevant cells constituting surrounding tissues (11,60-66) compared with the relatively less efficient nonviral vectors, the more immunogenic adenoviral vectors that also show very short-term gene expression capability, and the retroviral vectors that require division and pre-selection of the target cells for practicability (7). Another important point to consider in a human gene therapy trial is the ability of retroviral vectors to integrate into the host genome that might possibly lead to events of insertional mutagenesis and undesirable activation of tumor genes. Again to their advantage, rAAV vectors have been shown to be mostly maintained under stable episomal forms (67).

Key principles applied for gene-based approaches include the stimulation of anabolic pathways to enhance chondrogenesis, eg, cell proliferation and synthesis of extracellular matrix and the inhibition of catabolic pathways to prevent degradation (Table 1) (7). Among the gene candidates of value for articular cartilage repair, inhibitors of both matrix-degrading enzymes (tissue inhibitor of metalloproteinases) (9,68) and of proinflammatory cytokines (IL-1Ra, the soluble receptors sIL-1R, or sTNFR) (22,35,40,51,70), as well as chondroprotective cytokines (IL-4 and -10) (19,49,71) have been applied to inhibit catabolic pathways in vitro that are potentially activated in response to cartilage damage or injury. More relevant for the induction of cartilage repair, single or combined administration of components of the cartilage matrix or of the enzymes that synthesize them (77,78), of growth factors and their receptors (IGF-I, FGF-2, BMPs, TGF-β) (12,19,20,23,28-30,32,35,36,45,72,73), and of transcription factors (SOX5, SOX6, SOX9) (15,21,34,44,56,57) was successfully employed to activate anabolic processes in vitro. Alternatively, restoration of cell vitality and activation of proliferation in vitro have been achieved by application of IGF-I and FGF-2 (9,12,20,45,57,72), telomerase (hTERT) (75), inhibitors of apoptosis (bcl-2) (74), or HSP70 (16). Interestingly, approaches that influence several of these processes have been also successfully attempted, like combining the transfer of inhibitors of catabolism pathways and of activators of anabolic events (IGF-I/IL-1Ra or IGF-I/IL-4) (19,35,36), as well as that of activators of anabolic and proliferative processes (FGF-2/SOX9 or FGF-2/IGF-I) (57).

**Table 1:** Principles, mechanisms of action and gene candidates for articular cartilage repair

Mechanism of action	Target gene	References
**Inhibition of catabolic pathways**		
inhibition of matrix-degrading enzymes	TIMP	(68,69)
inhibition of proinflammatory cytokines	IL-1Ra, sIL-1R, sTNFR	(22,35,40,51,70)
chondroprotective cytokines	(IL-4, IL-10)	(19,49,71)
**Stimulation of anabolic pathways**		
growth factors	IGF-I, FGF-2, BMPs, TGF-β	(12,19,20,23,28-30,32,35,36,45,72,73)
chondrogenic transcription factors	SOX5, SOX6, SOX9	(15,21,34,44,56,57)
**Cytoprotection/** **proliferation**		
growth factors	IGF-I, FGF-2	(9,12,20,45,57,72)
inhibition of apoptosis	bcl-2, HSP70	(16,74)
catalytic component of human telomerase	human telomerase	(75)
**Combinatorial approaches**		
inhibition of catabolic pathways plus activation of anabolic pathways	IGF-I/IL-1Ra; IGF-I/IL-4	(19,35,36)
activation of anabolic plus proliferative pathways	FGF-2/SOX9; FGF-2/IGF-I	(57,76)

*TIMP – tissue inhibitor of metalloproteinases.

## Transplantation of genetically modified cells onto normal articular cartilage explants in vitro

**Figure 1:**
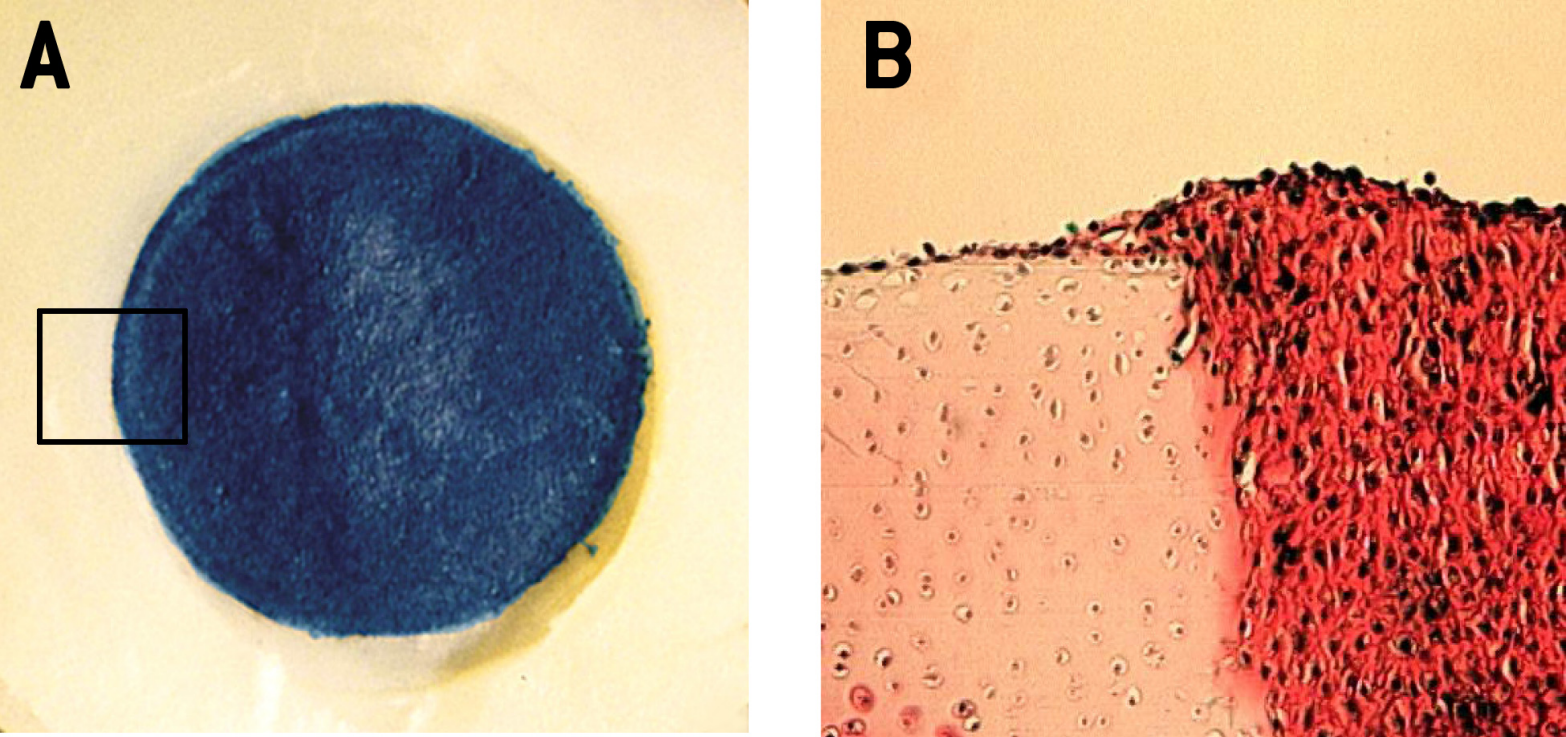
Macroscopic view (**A**) and histologic section (**B**) of a composite culture/ex vivo articular chondrocyte transplantation model of a chondral (partial-thickness) defect. (**A**) Bovine articular chondrocytes were transfected with the *lacZ*-plasmid vector pCMVßgal and transplanted into the partial-thickness chondral defect created in a normal bovine cartilage explant. The borders of the circular chondral defect can be easily identified. The transplanted chondrocytes express the transgene on day three post-transfection which can be identified by the intensive blue color. (**B**) Photomicrograph of the integration site of the new tissue that has formed based on the transplantation of ex vivo genetically modified chondrocytes with the neighboring articular cartilage. Note the good integration of these two tissues, the relative hypercellularity of the new repair tissue based on the genetically modified cells in comparison with the neighboring articular cartilage, which exhibits a loss of safranin O staining at his edge toward the defect as a result of the surgically induced lesion. Original magnification ×100 (**B**).

Transplantation of genetically engineered articular chondrocytes onto articular cartilage explants allows for the repopulation of articular cartilage and the creation of chimerical articular surfaces in vitro (12,20,70,79,80) (Figure 1). A prerequisite for such a transplantation approach is the adherence and integration of the cells to the articular surface with continuous expression of the transgene. Enzymatic treatment of the articular surface results in an intimate integration of the transplanted cells with the articular cartilage, facilitating the formation of the interface between the two tissues (20,81). Transgenes have been shown to be active for at least 2 weeks in vitro, a time frame sufficient to allow for structural changes. While researchers usually transplant cells that have been genetically modified, Doherty et al have demonstrated that transplanted articular chondrocytes are amendable as late as 35 days after transplantation to adenoviral gene transfer in vitro (79).

The new tissue that forms is characterized by the presence of type-II collagen and proteoglycans and by the absence of type-I collagen. In this model, transplantation of chondrocytes overexpressing BMP-7 resulted in a thicker new tissue layer (80). Overexpression of a human IGF-I cDNA by genetically engineered articular chondrocytes simultaneously promoted chondrogenesis and shifted the cartilage homeostasis toward an anabolic direction, as demonstrated by the increased rates of cell proliferation and of extracellular matrix synthesis in the new tissue layer (20). Remarkably, synthesis of DNA and of glycosaminoglycans was also stimulated in the underlying explant cartilage. When fibroblast growth factor 2 (FGF-2), a mitogen for chondrocytes, was applied by transfected chondrocytes, the proliferative activities were stimulated in the new tissue without effect upon matrix glycosaminoglycan synthesis (12). These data suggest that therapeutic FGF-2 gene transfer may be applicable for the treatment of cartilage defects when cellular repopulation is the therapeutic goal, while BMP-7 may have value to enhance matrix synthesis and IGF-I for circumstances demanding a balanced increase in both chondrocyte proliferation and matrix synthesis.

Gene transfer of transcription factors has received recent interest because of the impact of these molecules on chondrogenesis. Overexpression of SOX9 via retroviral transduction was shown to stimulate type-II collagen expression in pellet cultures of human osteoarthritic articular chondrocytes (44) and in human osteoarthritic articular chondrocytes embedded in alginate spheres (21,56,57).

## Transplantation of genetically modified cells to focal articular cartilage defects in vivo

In this section, a number of recent studies in the field of articular cartilage repair are highlighted, with an emphasis on preclinical approaches that appear to hold most promises for a possible clinical translation.

**Figure 2:**
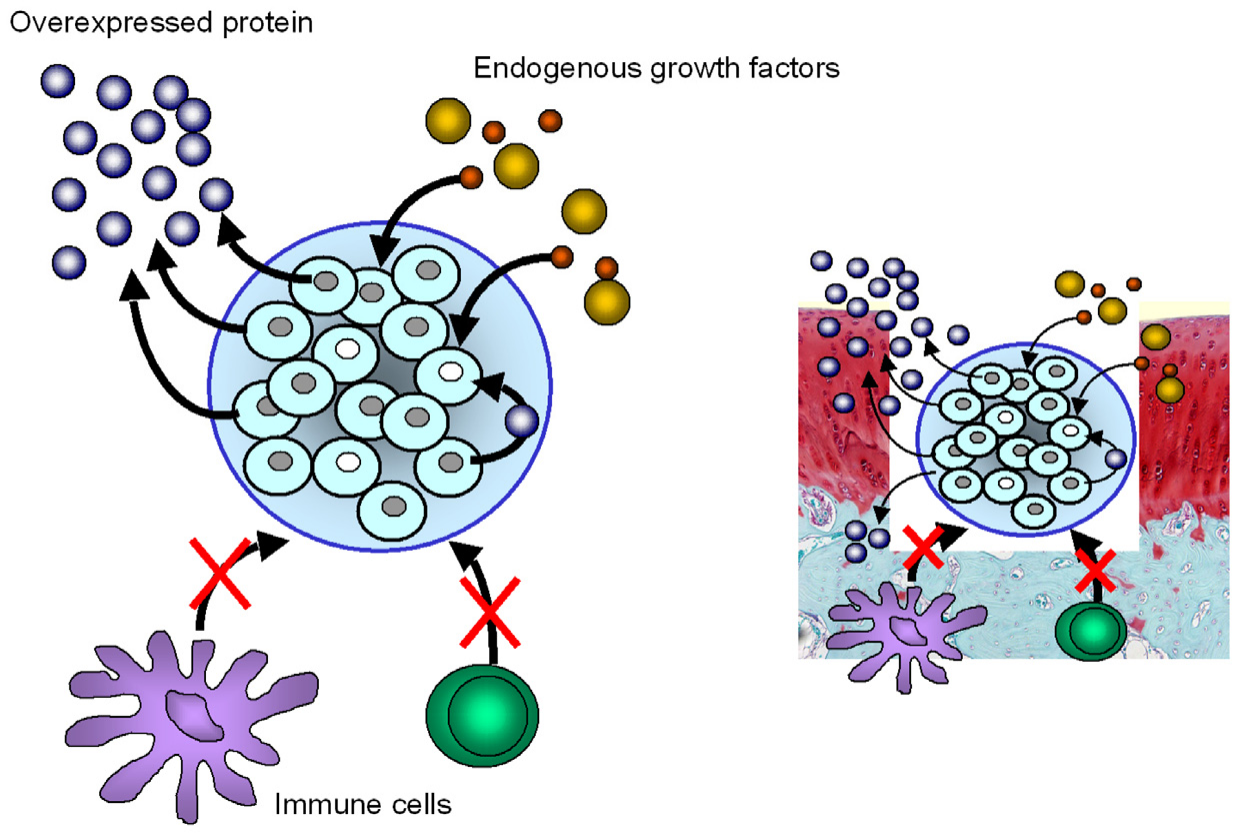
Principle of alginate encapsulation of transfected, genetically modified articular chondrocytes. The alginate beads are implanted in a subchondral location. Overexpression of a therapeutic transgene results in autocrine and paracrine signals targeting the cells within the alginate sphere. When the protein is secreted, paracrine effects may stimulate the repair tissue that forms in the defect, the neighboring normal articular cartilage and, via the synovial fluid, other cells of the joint such as cells of the synovial lining and periosteal cells. In parallel, the transplanted cells are also stimulated by factors from the host. The alginate sphere is thought to protect the transplanted cells from immune responses.

Transplantation of genetically modified cells into articular cartilage defects in vivo has been performed in three-dimensional systems, including cell aggregates such as coagulated bone marrow aspirates (82), by encapsulation of genetically engineered cells in supportive biomaterials like alginate (9,10,83) (Figure 2), agarose (84,85), fibrin or type-I collagen gels without (86-88) or with a periosteal flap (48,89), synthetic biodegradable scaffolds (90-92), and as genetically modified tissue-engineered cartilage (7). Kang et al were the very first to transplant chondrocytes genetically modified by a retroviral vector into an articular cartilage defect in vivo (93). Others used nonviral (94-96), adenoviral (70,82,87), retroviral (93,97-100), and rAAV vectors (101) to deliver reporter genes to defects via ex vivo approaches. While articular chondrocytes are usually transplanted (87,93,94,96,100,102,103), fibroblasts (76,104), perichondrial (95), periosteal (97,101), muscle-derived cells (98), or NIH 3T3 cells (76) have been also applied. Animal models for these marker gene studies included rats (87), rabbits (9,10,48,76,82-84,86,88,90-101,103,104), goats (89), and horses (102).

**Figure 3:**
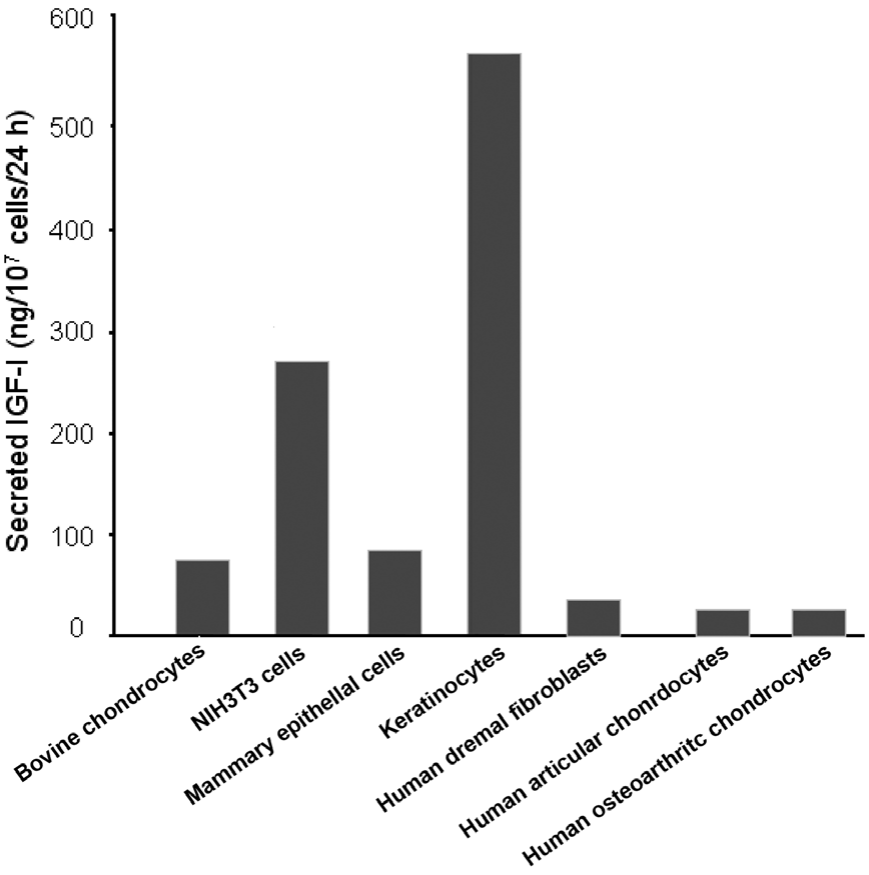
Secretion of human insulin-like growth factor I by bovine articular chondrocytes and NIH 3T3 cells transfected with FuGENE 6, by transduced bovine mammary epithelial cell line and by keratinocytes, both after retroviral transduction and selection, by human dermal fibroblast transduced with a retroviral vector, and by human normal and human osteoarthritic articular chondrocytes transduced with an rAAV-hIGF-I.

A prerequisite for the transplantation of genetically modified cells for articular cartilage repair is their capability to secrete pharmacologically active amounts of the recombinant protein (7). Yet, the cell type used for transplantation, the efficiency of the gene delivery method, and the possible influence of the biomaterial used for cell encapsulation critically affect the amount of a therapeutic protein that is produced and secreted (Figure 3). For example, IGF-I production by bovine articular chondrocytes transfected with a lipid-based method was reported to be 83 ng/10^7^ cells/24 hours (20), while 92 ng IGF-I/10^7^ cells/24 hours were secreted by a retrovirally transduced bovine mammary epithelial cell line selected for cells carrying the transgene (105), less than the 560 ng IGF-I/10^7^ cells/24 hours produced by keratinocytes after retroviral transduction and selection (106). Normal human dermal fibroblasts similarly retrovirally transduced secreted 39 ng IGF-I/10^7^ cells/24 hours but 214 ng IGF-I/10^7^ cells/24 hours when encapsulated in alginate microspheres (107). When human normal and osteoarthritic articular chondrocytes were transduced with an rAAV IGF-I vector, 27 and 19 ng IGF-I/10^7^ cells/24 hours were secreted (our unpublished data).

**Figure 4:**
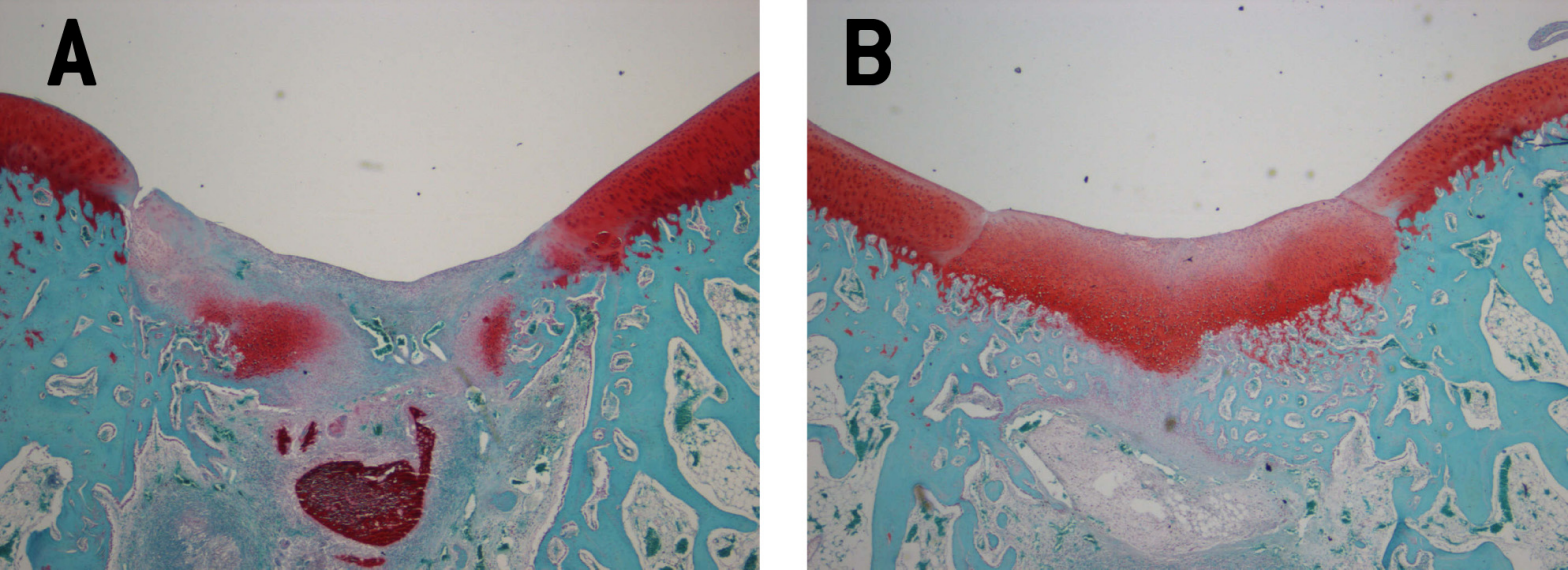
Cartilage repair three weeks after transplantation of genetically modified NIH 3T3 cells into an osteochondral defect model in the trochlea grove of rabbits. (**A**) Defect treated with the *lacZ* gene as a control. Note the remnants of the alginate sphere at the bottom of the defect. The repair tissue is largely negative for safranin O staining, indicating a lack of proteoglycans. (**B**) Contralateral knee of the same animal treated with the IGF-I gene. Although the margins of the defect can be still distinguished, a repair tissue rich in proteoglycans has been formed. Original magnification ×40.

Transgenes expressed via such ex vivo strategies remained active for about one month in the cartilage defects, significantly longer compared with the application of recombinant proteins. One of the earliest examples was provided by Mason et al who transferred the BMP-7 growth factor gene into a focal defect using periosteal cells transduced with a retroviral vector attached to a polyglycolic acid scaffold (39). As shown in this study, articular cartilage repair was improved at 8 and 12 weeks in vivo. A variety of therapeutic genes like BMP-2, BMP-7, IGF-I, FGF-2, and TGF-β (7,9,10,76,108-113) has been studied so far, with significant improvements in articular cartilage repair (7,9,10,76,108,110-113) (Figure 4). The choice of therapeutic genes is based on the strategy selected for improving articular cartilage repair. This, in theory, implies the use of genes that either stimulate chondrogenesis or inhibit articular cartilage degeneration. While the first approach has been studied in numerous investigations and is later described, the latter approach has been mainly tested in model systems of osteoarthritis so far. More specifically, strategies for improving individual parameters of chondrogenesis can be tailored. For example, IGF-I, a factor that simultaneously stimulates type-II collagen and proteoglycan synthesis as well as cell proliferation, may be of interest for strategies aiming at a balanced enhancement of these parameters (20), while FGF-2, which is mitogenic, may be useful to increase the pool of cells in a cartilage defect responsive to chondrogenesis (12). Initially, most of the evaluations were carried out in small animal models, such as rats (108,112) and rabbits (9,10,76,110,113). Hidaka et al (102) and Goodrich et al (114) performed the implantation of chondrocytes overexpressing BMP-7 or IGF-I in horses, respectively. Allogeneic chondrocytes transduced by an adenoviral vector carrying BMP-7 implanted into defects allowed for a better appearance of the repair tissue after 4 weeks, although no differences were found between the groups after 8 months (102). Goodrich et al showed that arthroscopically-grafted chondrocytes genetically modified by an adenovirus vector encoding equine IGF-I significantly increased IGF-I mRNA and ligand production in repair tissue in an equine model for up to 9 weeks following transplantation (114). Collagen type II expression in IGF-I treated defects was significantly increased and correlated with increased collagen type II immunoreactivity. In addition, genetic modification of chondrocytes prior to transplantation improved early (4 to 9 weeks), and to a lesser degree long-term, cartilage repair over control defects in vivo (114). Others used genetically engineered MSCs overexpressing growth factors such as BMP-4-transduced cells provided with fibrin glue in full-thickness chondral defects in rabbits (115). Histologic repair was significantly improved in defects receiving cells modified to overexpress BMP-4 compared with those where they carried a marker gene. Also, MSCs overexpressing TGF-β1 seeded into polylactide scaffolds improved extracellular matrix formation, reconstitution of the subchondral bone, and inhibited inflammatory responses (116). Finally, transplantation of MSCs transfected with the CDMP1 gene also enhanced the repair of osteochondral defects (117).

More recently, Ivkovic et al (118) implanted autologous bone marrow clots containing ex vivo adenovirally-transduced cells with a TGF-β1-expressing vector into partial-thickness chondral defects in sheep. Remarkably, improved histological, biochemical, and biomechanical parameters were evident in this preclinical animal model 6 months postoperatively.

Taken together, the current literature supports several conclusions illustrating the recent progress that has been made in developing approaches for the transplantation of genetically modified cells (eg, articular chondrocytes and MSCs) as a therapeutic option for human articular cartilage defects. First, there is convincing proof of principle that articular chondrocytes and MSCs modified by different nonviral and viral approaches can be transplanted into articular cartilage defects in vivo, allowing for sustained and clinically relevant transgene expression levels. Second, overexpression of therapeutic proteins enhances the structural features of the repair tissue of the articular cartilage and subchondral bone, being superior to unmodified or mock-transduced/-transfected cells. Third, combined overexpression of genes with complementary mechanisms of action (eg, chondrogenic and proliferative factors) is feasible, holding promises for further enhancement of articular cartilage repair. Fourth, significant benefits have been observed in preclinical animal models that are, in principle, more appropriate to study the situation in patients.

## Transplantation of genetically modified cells onto osteoarthritic cartilage explants in vitro

**Figure 5:**
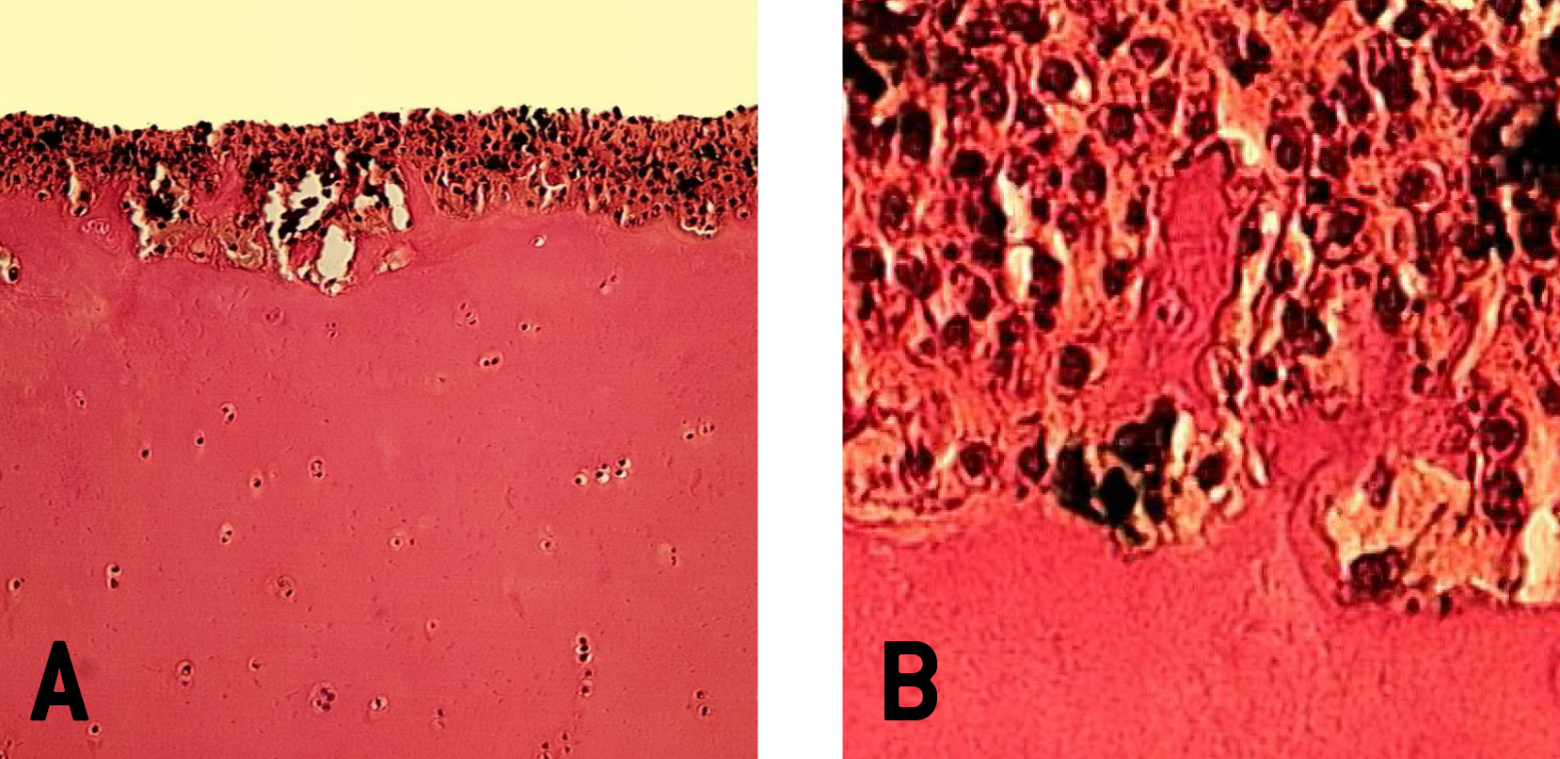
Photomicrograph of human osteoarthritic cartilage after transplantation of genetically modified bovine articular chondrocytes. Articular chondrocytes transfected with the *lacZ* gene have been seeded onto the surface of early stage human osteoarthritic cartilage. Note how the transplanted chondrocytes are filling the voids between the fragmented cartilage of the surface. Foci of transplanted chondrocytes expressing the transgene are identifiable by their blue color. (**A**) Overview and (**B**) high magnification of the integration between the newly formed tissue based on genetically modified chondrocytes and the original osteoarthritic surface. Safranin O-fast green. Original magnification ×100 (**A**) and ×200 (**B**).

Ex vivo modified cells may be also applied for the resurfacing of the disrupted cartilage matrix in early stages of osteoarthritis. When transplanted onto the surface of osteoarthritic human cartilage, the modified chondrocytes may fill the gaps between the fissures, cracks, and other surface discontinuities (Figure 5). Since osteoarthritis is characterized in part by the activation of inflammatory and catabolic processes, such pathways might be inhibited by applying chondrocytes, MSCs, or synoviocytes overexpressing inhibitory molecules, such as IL-1Ra alone (70,119-124) or combined with IL-10 (124).

Baragi et al were the first to show that transplantation of human osteoarthritic chondrocytes transduced with adenoviral vectors to overexpress the human IL-1Ra onto the articular surface of osteoarthritic cartilage explant cultures was capable of protecting the cartilage from IL-1-induced extracellular matrix degradation (70). Remarkably, transduced, transplanted chondrocytes secreted significant levels of biologically active IL-1Ra that were sufficient to allow for a resistance to IL1-induced proteoglycan degradation over the 10-day culture.

We transplanted bovine articular chondrocytes transfected with a nonviral vector overexpressing the *E.coli lacZ* gene onto the articular surface of human osteoarthritic cartilage explant cultures (Figure 5). Transfected, transplanted chondrocytes settled into the clefts of the osteoarthritic cartilage and restored a relatively even articular cartilage surface populated by the transplanted cells over the 7-day culture.

## Transplantation of genetically modified cells in models of osteoarthritis in vivo

Transplantation of genetically modified cells in experimental models of osteoarthritis in vivo has been mostly carried out by intra-articular injection of genetically modified cells, an approach also employed in experimental models of rheumatoid arthritis (125).

Pelletier et al (123) were among the first groups to inject retrovirally-modified synoviocytes to overexpress an IL-1Ra in a model of osteoarthritis in dogs, showing the successful reduction of the progression of the experimentally induced lesions. The same IL-1Ra gene sequence, provided alone or in combination with IL-10 via synoviocytes transduced with a retroviral vector was of further benefit to treat osteoarthritic in rabbits in vivo (124). More recently, Matsumoto et al (126) reported the efficacy of injecting intra-articularly muscle-derived stem cells modified by combined gene transfer of BMP-4 with sFlt1 (a vascular endothelial growth factor antagonist) for cartilage repair in a rat model of osteoarthritis.

In summary, there is a scarcity of experimental studies of transplanting genetically modified cells in models of osteoarthritis in vivo and, most importantly, none of the approaches described here for the treatment of osteoarthritis have been evaluated in large animal models to date.

## Clinical gene therapy trials using genetically modified cells

Preclinical data, as those described above, have encouraged the initiation of human clinical trials originally for arthritis, mostly based on the transfer of an IL-1Ra gene sequence (1,2,127-136) and of a sTNFR fusion protein (129,130,132,135,137-140) in the joints of patients with rheumatoid arthritis. Proof of concept and safety has been already established in the phase I clinical gene therapy study by Chris Evans in the USA and Peter Wehling in Germany (2). Autologous synovial fibroblasts transduced with a retroviral vector encoding for an IL-1Ra cDNA were injected into the 2nd-5th metacarpophalangeal joints of 9 postmenopausal women with advanced rheumatoid arthritis. Elevated amounts of IL-1Ra were present in the synovial fluid of treated patients, and cells expressing high levels of IL-1Ra were found in the synovial membrane. There were no adverse events. This investigation highly encouraged further development of gene-based approaches for the treatment of cartilage disorders. Regarding osteoarthritis, a phase I protocol is currently ongoing, based on an ex vivo approach using the retroviral transfer of transforming growth factor (TGF)-beta (130).

## Challenges for the clinical application of transplanting genetically modified cells to promote articular cartilage repair

In conclusion, transplantation of genetically modified cells is a promising tool for the treatment of articular cartilage defects such as resulting from trauma or osteoarthritis. In the past decades, considerable progress has been made to lay the scientific foundation for the use of modified cells. In particular, articular chondrocytes and, more recently, MSCs have been genetically modified using nonviral or viral methods. When used in model systems of cell transplantation, structural improvements occurred in the new repair tissue. Moreover, in small animal models, transplantation of genetically modified cells has yielded significant structural improvements of the repair tissue. Lately, preclinical animal models have been applied to study the effects of overexpression of therapeutic genes (118). However, to realize the full potential of these approaches in the clinics, the following issues have to be addressed:

1. the proof of the safety of this approach in preclinical and clinical trials

2. the choice of the ideal gene vector system

3. the choice of the optimal therapeutic gene(s)

4. the delivery without (eg, for large osteoarthritic lesions) or with a supportive biomaterial (for focal lesions),

5. the establishment of the optimal cell dose.

For traumatic cartilage defects, single or combined overexpression of therapeutic genes, including growth and transcription factors, is feasible and results in structural improvements. Significant data have been collected in animal models of chondral and osteochondral defects. For osteoarthritis, transplantation approaches using genetically engineered cells have been less developed. Ex vivo models have already convincingly shown that genetically modified chondrocytes adhere to human osteoarthritic cartilage explants, and that inhibition of matrix degradation is possible. Animal studies using genetically modified cells for the treatment of osteoarthritis have mainly relied on the intra-articular injection of these cells, making a specific targeting of osteoarthritic areas difficult. Moreover, it remains to be seen whether areas of cartilage loss may be repopulated by such approaches, considering that the nature of the osteoarthritic loss of articular cartilage is unknown and since a simple injection of cells alone does not address other factors involved in the pathogenesis of osteoarthritis such as mechanical overload induced by axial malalignment.

Several gene transfer systems are capable of successfully modifying articular chondrocytes and MSCs, the two most promising cell types for transplantation approaches. However, each of the individual vector system has intrinsic limitations with regard to their efficacy (nonviral systems) and safety (eg, adenoviral systems) for human clinical applications. Most experts predict that initial human gene therapy trials based on the transplantation of genetically modified cells for articular cartilage repair will be conducted using rAAV vectors. The development of novel (third generation) adenoviral vectors may be another option, although significantly less immunogenic responses might be elicited when using rAAV. Vectors showing a capacity for site-specific integration have been designed for the purpose of achieving long-term gene expression, such constructs have not been tested yet in articular cartilage repair settings.

Supportive biomaterials may be used to deliver the genetically modified cells to focal articular cartilage defects, similar to the current clinical practice of treating cartilage lesions with matrix-assisted autologous chondrocyte transplantation, a technique in which the articular chondrocytes are attached to different types of scaffolds, eg, based on collagen types I/III, hyaluronic acid, polyglactin/poly-p-dioxanon, and fibrin-hyaluronan (141). Such a delivery of genetically modified chondrocytes in conjunction with biomaterials may be advantageous compared to cell delivery alone, as it allows for a spatially controlled application of the modified cells to enhance chondrogenesis. An overexpression of therapeutic genes in such tissue-engineered constructs offers the important advantage of a complete filling of the defect with a construct actively supporting chondrogenesis. Although possible side-effects intrinsic to the biomaterial on transgene expression have to be ruled out in experimental studies, the current clinical consensus on the broad advantages of matrix-assisted autologous chondrocyte transplantation suggest that genetically modified cells may rather be delivered in supportive biomaterials to focal cartilage defects. For the treatment of osteoarthritis, however, no conclusions can be drawn at the moment because of the paucity of experimental preclinical and clinical in vivo data.

Possible side-effects have also to be considered. While intra-articular injection of an BMP-2 adenoviral vector resulted in the formation of osteophytes and spread of the vector DNA to the liver, lung, and spleen, no such effects were observed when an ex vivo approach was selected injecting modified cells overexpressing BMP-2 instead (31). In fact, it appears that the therapeutic protein may exert its role in close proximity to the defect, as judged by the absence of elevated protein levels in the synovial fluid and peripheral blood and by the detection of only a few days of marker gene activity in cells adjacent to the site of the surgical approach.

The selection of therapeutic genes to specifically address individual parameters of chondrogenesis will continue to be a challenge. Progress may come from more insights into the regulation of chondrogenesis, eg, using genomic and proteomic profiles.

Open questions remain also in terms of finding the optimal cells dose. In the clinical protocols of autologous chondrocyte transplantation, chondrocytes are usually transplanted at densities of about 1 × 10^6^ cells/cm^2^. When genetically modified articular chondrocytes overexpressing IGF-I were applied in a lapine model, about 75 × 10^6^ cells/cm^2^ were applied, a 75-fold higher dose (10). In the clinical gene therapy study by Chris Evans, 1 × 10^6^ (low dose), 1.5-5 × 10^6^ (intermediate dose), and 6.5-10 × 10^6^ (high dose) autologous synovial fibroblasts transduced with a retroviral vector encoding for an IL-1Ra cDNA were injected into metacarpophalangeal joints (2). Because of its similarity with the protocol currently used for autologous chondrocyte transplantation, it is likely that a dose of 1 × 10^6^ cells/cm^2^ may be elected as a starting point of evaluation when genetically modified chondrocytes will be transplanted to focal cartilage defects in patients.

Nevertheless, nearly three decades of experimental and clinical research to advance the principles of articular chondrocyte transplantation have yielded significant technical improvements of clinical articular chondrocyte transplantation, such as chondrocyte application in highly specialized scaffolds for optimal cell attachment and distribution in vivo (141). Many of the procedures required to constitute such ex vivo gene transfer approaches are already available for the orthopaedic surgeon. For example, cell (chondrocyte) isolation and in vitro cell culture – essential steps of such ex vivo protocols – are already in place. Moreover, the problem of the loss of the chondrocytic phenotype – as observed after prolonged monolayer culture (142), has been sufficiently addressed. These autologous chondrocytes could easily be genetically modified ex vivo – as shown, for example, for hematopoietic stem cells (143) – and could be implanted at the site of the cartilage defect, where a high concentration of the chondrogenic agent is needed. For the transplantation of MSC it remains to be seen whether the terminal differentiation of chondrocytes when they become hypertrophic – as observed in vitro – represents indeed a problem when implanted into articular cartilage defects in vivo (144,145).

Despite these encouraging data, application of genetically modified cells to treat cartilage defects is still not on the horizon. Issues that need to be addressed include the duration of transgene expression, extended studies with both nonviral and viral transfer systems in preclinical models of focal and osteoarthritic cartilage defects, the continuing elucidation of the benefit of using ex vivo genetically modified cells vs direct gene transfer approaches, and the ongoing identification of optimal therapeutic factors. Future studies will also have to prove the safety of such approaches, since both traumatic defects and osteoarthritis are non-lethal diseases.

A key challenge in combining both experimental (in the case of genetic modification) and clinical knowledge (in the case of articular chondrocytes transplantation) and, as a result, to translate these progresses into clinical medicine is finally to establish a safe and efficient production line, including vector manufacturing, cell isolation, and genetic modification that meets all regulatory requirements.

Ultimately, the clinical potential for genetically modified articular chondrocytes and MSCs for the treatment of articular cartilage defect is likely to be realized by advances in the following areas: 1) development of a safe, highly efficient gene delivery system with sustained duration of transgene expression, 2) identification of optimal therapeutic gene(s), 3) combination of genetically modified articular chondrocytes and/or MSCs with scaffolds that better support chondrogenesis in vivo. After safe techniques for generating these genetically engineered cells are available, such cells may eventually provide new avenues for improved cell-based therapies for articular cartilage repair. This, in turn, may provide a crucial step toward the unanswered question of articular cartilage regeneration.
